# Recurrent squamous cell carcinoma and a novel mutation in a patient with xeroderma pigmentosum: a case report

**DOI:** 10.1186/s13256-022-03524-2

**Published:** 2022-07-28

**Authors:** Ezgi Aysu Şahin, Ekim Zihni Taşkıran, Pelin Özlem Şimşek Kiper, Burça Aydın, Eda Utine

**Affiliations:** 1 Hacettepe University, Faculty of Medicine, Ankara, Turkey; 2 Department of Medical Genetics, Gene Mapping Laboratory, Hacettepe University Medical Faculty, Ankara, Turkey; 3 Department of Pediatrics, Pediatric Genetics, Hacettepe University Medical Faculty, Ankara, Turkey; 4 Department of Pediatric Oncology, Institute of Oncology, Hacettepe University, Ankara, Turkey

**Keywords:** Xeroderma pigmentosum, Squamous cell carcinoma, Whole-exome sequencing, Immune checkpoint inhibitors, Nucleotide excision repair, Case report

## Abstract

**Background:**

Xeroderma pigmentosum is an extremely serious genetic disorder defined by sensitivity to sunlight, resulting in sunburn and pigment changes. If patients are not completely protected from ultraviolet radiation, xeroderma pigmentosum is characterized by a greatly increased risk of sunlight-induced cutaneous neoplasms. There is no standard therapy for skin cancer of xeroderma pigmentosum. However, immune checkpoint inhibitors were reported to increase response rates and improve outcomes and life expectancy in patients with various cancers, including squamous cell carcinoma in xeroderma pigmentosum. In this paper, we report on a patient with xeroderma pigmentosum from a consanguineous family with recurrent facial chemotherapy-resistant squamous cell carcinoma lesions treated successfully with an anti-programmed cell death protein 1 monoclonal antibody in both relapses.

**Case presentation:**

A 7-year-old Turkish male was referred to our oncology department for recurring squamous cell carcinoma after local excision of the tumor over his nose. The lesion was a rapidly growing lesion, measuring 8 × 4 cm in size. Physical examination revealed that he also had hemorrhagic crusted plaques and nodules over both eyelids and upper lip, with multiple hypo- and hyperpigmented punctate lesions all over his body. After two more cycles of chemotherapy, progressive disease was noted, and a new lesion on the right eyelid caused blurred vision. Anti-programmed cell death protein 1 antibody treatment was planned with concomitant radiotherapy. He received nivolumab every 3 weeks for 4 months, improving his vision. No new lesions or active complaints have been observed in the current situation, and complete remission has been achieved. On the last admission, the patient was clinically diagnosed with xeroderma pigmentosum. Owing to the condition’s genetic heterogeneity, whole-exome sequencing was performed with Ion Proton next-generation sequencing platform, and the c.2250 + 1G>A splice site mutation of the *XPC* gene was detected in the homozygous state.

**Conclusions:**

The clinical report emphasizes the importance of clinical awareness and crucial early diagnosis of xeroderma pigmentosum and presents a novel causative homozygous c.2250 + 1G>A splice site mutation. Our case proves that next-generation sequencing is an effective method for the rapid diagnosis and determination of xeroderma pigmentosum genetic etiology.

## Background

Xeroderma pigmentosum (XP) is a rare, inherited autosomal recessive genetic disorder defined by extreme sensitivity to ultraviolet radiation (UV), resulting from defective DNA repair [1, 2].

These patients have 1000-fold increased risk of developing early malignant neoplasms, primarily skin cancers [3]. XP affects males and females equally, but the incidence of the disorder varies among countries. Japan’s estimated incidence is 1 in 20,000 healthy children, while it is seen 1 in 250,000 in the USA. Approximately 2.3 per million live births are diagnosed with XP in Western Europe [2, 4].

XP is classified into eight types according to which gene is mutated, which normally function in repairing DNA damage induced by UV radiation by a process known as nucleotide excision repair (NER) [5, 6]. UV radiation causes the formation of photoproducts in DNA, which predisposes to carcinogenesis and the promotion of cellular aging, cell death, and mutagenesis [5].

Increased risk of cutaneous neoplasms in patients with XP mainly causes basal cell carcinoma, squamous cell carcinoma (SCC), and melanoma. Early diagnosis of the condition and extensive sun protection can prevent the development of skin cancers in patients with XP and increase life expectancy [3]. The XP diagnosis is based on clinical findings, as well as identification of biallelic pathogenic variants in *DDB2*, *ERCC1*, *ERCC2*, *ERCC3*, *ERCC4*, *ERCC5*, *POLH*, *XPA,* or *XPC* in family history [7]. Owing to the genetic heterogeneity of the condition, whole-exome sequencing (WES) is a practical and rapid method of molecular diagnosis in these cases [7].

In this paper, we report on a patient with XP and recurrent SCC treated successfully with an anti-programmed cell death protein 1 (anti-PD-1) monoclonal antibody in both relapses. A novel mutation, homozygous c.2250 + 1G>A splice site mutation, was detected in the *XPC* gene.

## Case presentation

**Fig. 1:**
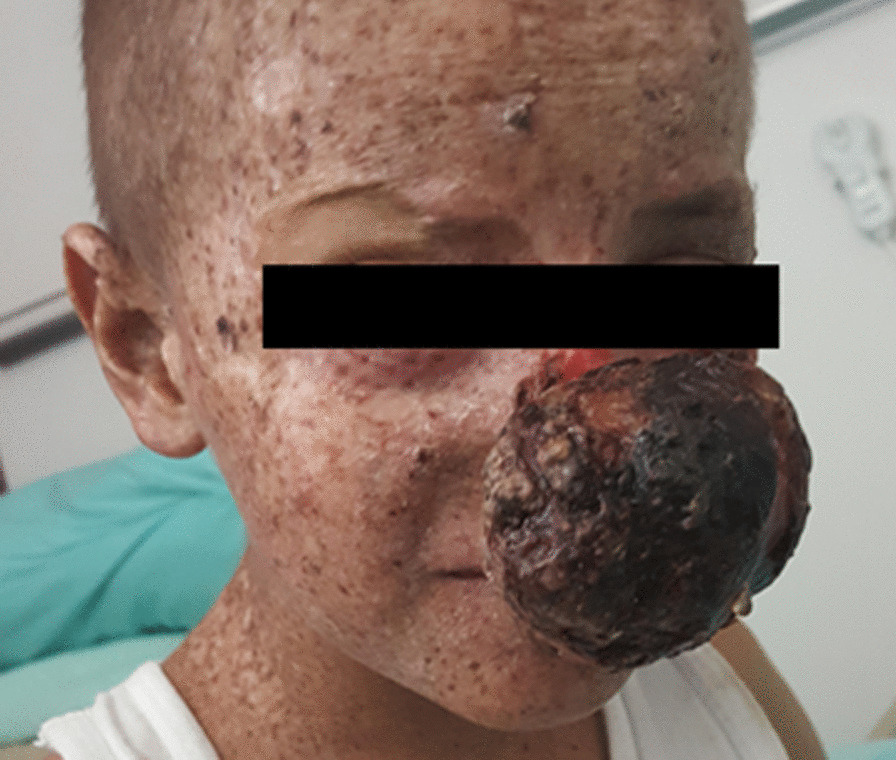
Clinical photograph showing a rapidly growing lesion on dorsum of nose and pigmented macules on sun-exposed regions of the body

With a clinical diagnosis of XP, a 7-year-old, Turkish male was referred to the Department of Oncology at Hacettepe University for the recurring SCC after local excision of the tumor over his nose. The lesion was a rapidly growing lesion, this time measuring 8 × 4 cm in size (Fig. 1). Physical examination revealed that he also had hemorrhagic crusted plaques and nodules over both eyelids and upper lip, as well as multiple hypo- and hyperpigmented punctate lesions all over his body.

Perinatal and early postnatal histories were unremarkable. He had no symptoms before 2 years of age when hyperpigmented spots were first recognized. The parents were first cousins. There was no history of other family members having a similar disorder. There was no known disease in his two siblings, either. The child was regularly immunized. The developmental milestones were normal. Systemic examination, including neurological functions, was essentially normal.

Starting at the age of 2 years, the patient had hyperpigmented spots all over his face and arms, more prominent on his forearm, and bilateral widespread hypopigmented areas over the thighs. This abnormal pigmentation progressively worsened on exposure to sunlight, particularly in overexposed areas. With these complaints, the patient received a clinical diagnosis of XP. When he was 2 years old, a crusted erythematous lesion appeared on the edge of his left eye, as well. Following facial lesion excision, the pathology result was found to be consistent with SCC. After a year, lesions on one side of his nose and two other parts of his face also were excised and reported again as consistent with SCC (4 × 5 × 7 cm in nasal region, 1 × 2 × 5 cm in left zygomatic, 4 × 2 × 2 satellite lesion in maxilla).

**Fig. 2:**
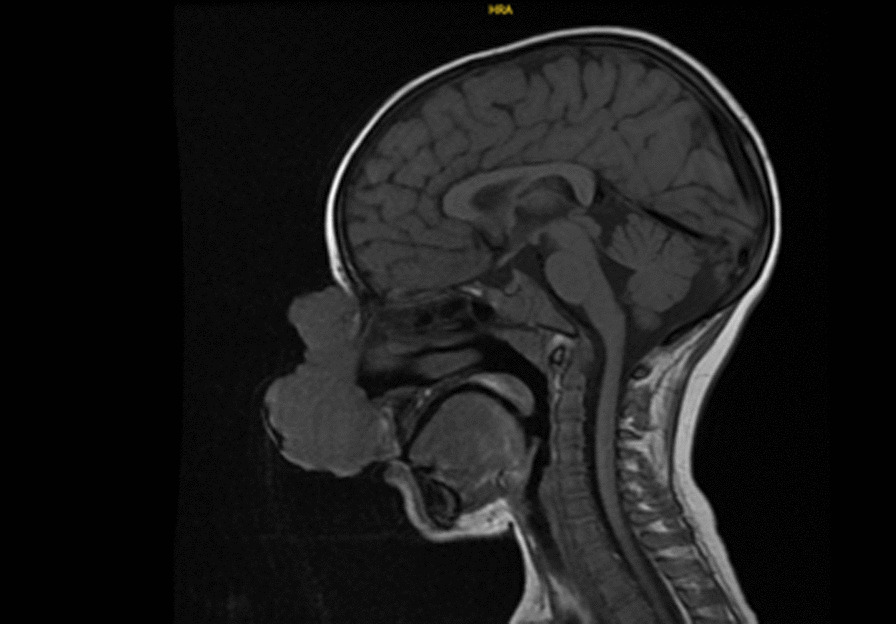
T1 sagittal magnetic resonance imaging of the brain; a lobulated giant mass that causes narrowing of the right nasal aperture with external pressure, located on the skin, starting from the level of the nasal root and extending to the level of the right nasal aperture

**Fig. 3:**
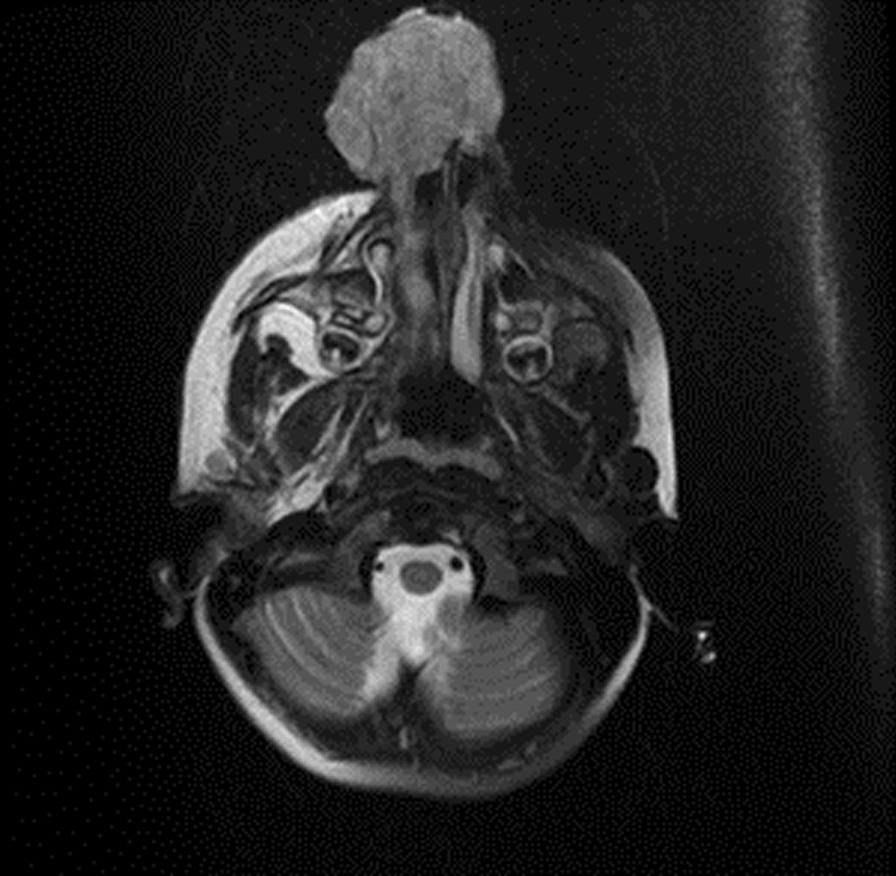
Brain magnetic resonance imaging T2 fat-saturated; a skin-derived lobulated mass with a vertical length of approximately 7.4 cm and transverse dimensions of 4.6 × 4.3 cm at the level of the right nasal aperture at its widest point is observed. Invasion of the mass into the nasal passage or ethmoid cells was not detected

At the age of 6 years, the patient was admitted to our hospital for a rapidly growing nasal lesion (Figs. 2 and 3). Incisional biopsy was done from the lobulated giant mass that caused narrowing of the right nasal aperture with external pressure. Pathological examination revealed SCC as being medium to well differentiated. The right submandibular and preauricular metastatic lymph nodes (LN) were noted during the postoperative period. Chemotherapy was started with reduced doses with cisplatin and 5-fluorouracil owing to metastatic LN and unresectable primary tumor. Surgical excision could be performed after six cycles of chemotherapy, and the large mass of 9.5 cm in longest diameter was excised from the nose with positive surgical margin. After two more cycles, progressive disease was noted with enlarged submandibular LN and a new lesion on the right eyelid causing blurred vision. Anti-PD-1 antibody (nivolumab) treatment was planned with concomitant radiotherapy (66 Gy to the primary nasal mass, 60 Gy to preauricular LN, and 54 Gy to bilateral neck). He received nivolumab every 3 weeks for 4 months, and his vision was improved. However, after 4 months, nivolumab had to be stopped owing to financial reasons. Control magnetic resonance imaging (MRI) showed improvement in LN, and no new skin lesion was detected. He was followed up without any systemic chemotherapy, but local fluorouracil cream was used for new superficial lesions that appeared on the scalp, tongue, and right auricle.

After 18 months of follow-up, relapse was observed on a new lesion on his nape. The excised lesion was diagnosed as recurrent SCC. Nivolumab was planned, but owing to refusal of financial approval of nivolumab, paclitaxel (weekly 80 mg/m^2^) was given for 5 months. Partial response was noted in periorbital lesions, but auricular lesions were stable. Nivolumab was approved and was given for 15 months. No new lesions or active complaints have been observed in the current situation, and complete remission has been achieved.

**Fig. 4:**
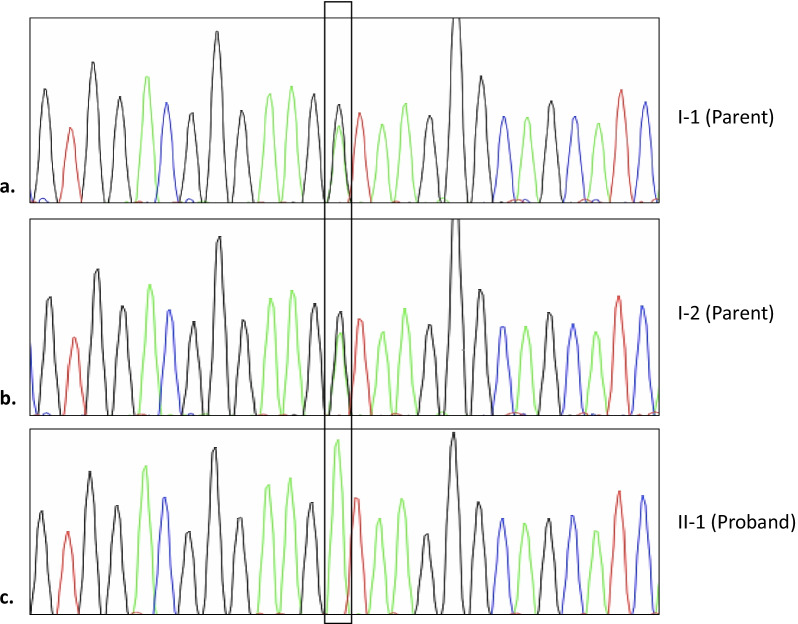
Sanger sequencing results in the proband’s family. According to the results, the parents of proband are both carriers of c.2250 + 1G>A (**a** and **b**), homozygous in the patient (**c**)

Owing to the condition’s genetic heterogeneity, WES was performed with Ion Proton next-generation sequencing platform, and the c.2250 + 1G>A splice site mutation of the *XPC* gene was detected in the homozygous state. The variant was then validated by Sanger sequencing (Fig. 4). This gene was associated with XP group C. This homozygous c.2250 + 1G>A splice site mutation is a mutation not yet reported in the ClinVar database. The parents of the patient were carriers (heterozygous) for the relevant mutation. The novel *XPC* variant identified in this study has been submitted to ClinVar with the submission ID of SUB11135062.

## Discussion

We herein present a patient from a consanguineous family with recurrent facial chemotherapy-resistant SCC lesions. The child was not diagnosed with XP until his last admission at 7 years of age, although he had typical salt-and-pepper appearance of the sun-exposed areas and UV-exposure related recurring SCCs. We emphasize that clinical recognition of this condition is possible and essential. WES is a practical tool for determining the underlying genetic defect in single-gene disorders with genetic heterogeneity and allowed for the definition of a novel splice site mutation, c.2250 + 1G>A, in the present patient.

Under the age of 20 years, the risk of nonmelanoma skin cancer has increased 10,000-fold, and that of melanoma has increased 2000-fold, in XP [8]. The most common cancers in patients with XP are BCC and SCC, mainly affecting the face, head, and neck [5]. Early detection of these malignancies is essential as they grow fast, metastasize in early phases, and even lead to death [9]. Accordingly, major causes of mortality in XP are metastatic malignant melanoma and SCC, caused by ultraviolet-induced skin hypersensitivity [10].

Children with XP develop multiple cutaneous malignancies at a young age [5], similar to our case, who developed SCC at the age of 2. Generally, significant tissue damage occurs by the time of diagnosis, and most untreated patients die before the age of 20 years [1]. XP is ultimately fatal, though life expectancy can be prolonged by minimizing sun exposure. Early diagnosis and treatment of these skin lesions and malignancies will reduce morbidity and mortality of the disorder [10].

Compared with the healthy population, patients with XP are at a several thousand-fold increased risk of skin cancer [1], and apart from skin cancers, there is also a 50-fold increased risk of systemic malignancy [11]. DNA damage plays a significant role in carcinogenesis by promoting cellular transformation [12]. Being an inherited disease, a radical cure today is unexpected in XP disorder, and cancer is the major cause of mortality and morbidity in these patients. There is no standard therapy for skin cancer of XP [13]. Chemotherapeutics might cause substantial organ toxicity and usually need to be used in reduced doses, which decrease the survival rates. Thus, immune checkpoint inhibitors were reported to increase response rates, maintain durable responses, and improve outcomes. They also might help prevent chemotherapy-related side effects if they were to be used at the first-line regimen. Our patient used six cycles of cisplatin–fluorouracil chemotherapy before surgical excision. Programmed death-ligand 1 (PDL-1) antibody nivolumab produces a complete response to progressive disease. One important feature of the patient was the complete response obtained with reused nivolumab after relapse. There was no resistance to the drug given for the second time, and the patient is well with no new lesion or any systemic sign of metastasis. Immune checkpoint inhibitors mainly target PD-1 and PDL-1, and they improve outcomes in many cancers, as well as SCC in XP [14].

XP means “dry pigmented skin” and is characterized by mucocutaneous and ocular hypersensitivity to UV radiation with irreparable DNA damage, and also by progressive neurological degeneration in some subjects [1], including cognitive impairment and progressive hearing loss [7]. Approximately 25% of patients with XP have progressive neurological degeneration leading to a shortened lifespan [2, 15]. Excessive sensitivity to sunlight result in severe sunburn and photophobia [11]. In addition to sunburn, the first finding in patients is usually lentiginosis (marked freckle-like pigmentation of the face) [1], which is first noticed around the age of 2, like in our patient, progressively settling in sun-exposed areas. Over time, the number of lentigines increases, and photoaging, xerosis, skin laxity, and poikiloderma (dyspigmentation) appear [1, 2, 7]. With multiple hypo- and hyperpigmented lesions all over his body, our patient was also affected with hemorrhagic crusted plaques and nodules over both superior eyelids. It is common for patients with XP, as the present patient, to have ocular abnormalities, such as photophobia and keratitis [2, 7], caused by ultraviolet-induced DNA alteration to epithelial cell conjunctiva, the cornea, and the eyelid [5, 7].

XP is a hereditary autosomal recessive disorder, with a prevalence of 1:1,000,000 in the USA and 1:100,000 in Japan [1]. The prevalence is higher in Middle Eastern countries such as Turkey, Israel, and Syria because of the high frequency of consanguineous marriages [11, 16]. Recent Turkish Demographic and Health Surveys reported the rate of consanguineous marriages as 22–24% in Turkey [17]. In a demographic study among patients with XP by Akdeniz *et al*. [16], the parental consanguinity rate was 86.7%, which is consistent with previous reports by Khatri *et al*. (92.8%) [18], Metin *et al*. (100%) [19], and Gül *et al*. (83.3%) [20]. The parents of the present patient were first cousins, and they were heterozygous carriers of the relevant mutation. Genetic counseling should emphasize the importance of avoiding further consanguineous marriages for prevention [21].

XP can result from mutations in any one of eight genes (*XPA*, *XPB*, *XPC*, *XPD*, *XPE*, *XPF*, *XPG*, and *XPV*). From *XPA* to *XPG*, the encoded proteins are involved in repairing UV-induced photoproducts in DNA by the process of NER [22]. The NER system is capable of repairing DNA damage resulting from UV radiation [23]. Each of these genes corresponds to clinically different complement subgroups. For example, neurological symptoms are observed in diseases caused by mutant *XPA*, *XPD*, and *XPG*, but not in others generally. Sunburn is also observed in *XPA*, *XPB*, *XPD*, and *XPG*, while not in the others [11]. However, it is still not possible to distinguish these subgroups on the basis of only clinical findings; mutation analysis is required. In the present patient, the c.2250 + 1G>A splice site mutation of the *XPC* gene was detected when WES analysis was performed. *XPC* is the most common complement type in the USA, Europe, and North Africa [24].

Over the past decade, WES has afforded an efficient and straightforward diagnosis method for patients with Mendelian disorders, and it has been helpful for inferring pathogenesis [25, 26]. While first-generation sequencing of all exons is difficult and time consuming [27], next-generation sequencing, specifically WES, is more efficient than first-generation sequencing approaches, with similar costs [26]. Besides offering a rapid and effective manner of genetic analysis, WES allows the detection of novel genes involved in pathogenesis [7, 21, 28]. In our case, WES analysis was performed with Ion Proton next-generation sequencing platform, and a novel pathogenic homozygous mutation in the *XPC* gene was identified.

## Conclusion

The present report emphasizes the importance of clinical awareness and crucial early diagnosis of XP and presents a novel causative mutation in *XPC* detected by WES. Although there is no cure for XP, early diagnosis of XP cutaneous disease and appropriate protection from sunlight would dramatically improve the patients’ quality of life and life expectancy. Also, checkpoint inhibitors might be included in the treatment of malignant skin cancers in XP, as in our patient treated successfully with an anti-PD-1 monoclonal antibody with recurrent chemotherapy-resistant squamous cell carcinoma lesions.

## Data Availability

Data sharing does not apply to this article as no datasets were generated or analyzed during the current study.

## Abbreviations

XP: Xeroderma pigmentosum
UV: Ultraviolet radiation
NER: Nucleotide excision repair
SCC: Squamous cell carcinoma
LN: Lymph nodes
WES: Whole-exome sequencing
